# SIRT1 and LSD1 competitively regulate KU70 functions in DNA repair and mutation acquisition in cancer cells

**DOI:** 10.18632/oncotarget.10328

**Published:** 2016-06-30

**Authors:** Mendel Roth, Zhiqiang Wang, Wen Yong Chen

**Affiliations:** ^1^ Department of Cancer Biology, Beckman Research Institute, City of Hope, Duarte, CA 91010, USA

**Keywords:** chronic myeloid leukemia, BCR-ABL, NHEJ, lysine deacetylase, lysine demethylase

## Abstract

Acquisition of BCR-ABL mutations underlies drug resistance of chronic myeloid leukemia (CML) to tyrosine kinase inhibitors, but the molecular mechanisms of mutation acquisition are poorly understood. We previously showed that lysine deacetylase sirtuin 1, SIRT1, promotes acquisition of BCR-ABL mutations in association with enhancing KU70 mediated non-homologous end joining DNA repair. In this study, we demonstrate that lysine specific demethylase 1 (LSD1) plays an opposite role to SIRT1 in regulating DNA repair and mutation acquisition. In response to therapeutic stress and DNA damage, LSD1 and SIRT1 compete for binding to KU70 on DNA damage foci globally and on the ABL locus. The recruitment of SIRT1 or LSD1 to KU70 impacts chromatin structure but does not correlate well with their direct histone modification functions, and SIRT1 helps maintain histone H4K16 acetylation and open chromatin for repair. The competitive KU70 binding by these proteins affects cancer cells' ability to repair broken DNA and acquire resistant genetic mutations in CML and prostate cancer cells. We identify that the core domain of KU70 binds both LSD1 and SIRT1, forming a molecular basis for the competition. The C-terminal SAP motif of KU70 mediates LSD1/SIRT1 competitive interaction by suppressing LSD1 binding to KU70 and ectopic expression of SAP-deleted KU70 to CML cells compromises their ability to acquire BCR-ABL mutations. Our study reveals a novel cellular stress response mechanism in cancer cells and a key role of LSD1/SIRT1/KU70 dynamic interaction in regulating DNA repair and mutation acquisition.

## INTRODUCTION

Transformation of hematopoietic stem cells by the BCR-ABL fusion oncogene leads to development of chronic myeloid leukemia (CML). Tyrosine kinase inhibitor imatinib mesylate (IM) is an effective treatment for the disease [1], but forfeits its efficacy in some patients, particularly those in advanced phases of the disease, due to acquired resistance through BCR-ABL mutations [2, 3]. To dissect the mechanisms of resistance, we previously developed a culture model with a blast crisis CML cell line that recapitulates clinical CML acquired resistance through BCR-ABL mutations [4]. Using this model, we showed that NAD^+^ dependent protein lysine deacetylase SIRT1 is critically involved in promoting acquisition of BCR-ABL mutations in response to IM treatment [5]. We also demonstrated that induction of cell differentiation by all-trans retinoid acid (ATRA) increases expression of cellular NAD^+^ cyclase CD38 that reduces cellular NAD^+^ concentration, inhibits SIRT1 activity and blocks BCR-ABL mutation acquisition [6].

SIRT1 is a multi-functional enzyme that deacetylates histones including H4K16 to regulate gene expression and many non-histone proteins for biological functions [7]. A key downstream effector of SIRT1 is KU70, a crucial factor for non-homologous end joining (NHEJ). NHEJ is a major DNA repair mechanism in mammalian cells for double strand breaks (DSBs) that can arise from intrinsic sources such as reactive oxygen species or from external sources such as cancer chemotherapeutic agents and ionizing radiation [8]. NHEJ repair is initiated when KU70/KU80 heterodimer binds to broken DNA ends. Both KU factors are essential for NHEJ as deletion of either one leads to DSB repair impairment and sensitivity to radiation [9, 10]. KU70 is subjected to lysine acetylation modification [11], and deacetylation of KU70 by SIRT1 stimulates KU70-mediated NHEJ repair [5, 12]. Besides its well-known function in NHEJ, KU70 has roles in non-DNA repair events, which are less understood. Among them, SIRT1 deacetylation of KU70 sequesters BAX protein in the cytoplasm to prevent apoptosis initiation and extend cell survival [13]. We have shown that SIRT1 promotes acquisition of resistant BCR-ABL mutations in CML cells in association with its ability to stimulate aberrant NHEJ activity by deacetylating KU70 [5, 6].

Lysine specific demethylase 1 (LSD1) is a monoamine oxidase homolog that demethylates histone H3K4 and H3K9 [14–16], and functions to regulate gene expression [17, 18]. LSD1 also demethylates non-histone proteins such as p53 for regulating cell survival [19]. Previously, we demonstrated that p53 deacetylation by SIRT1 plays a key role for drug resistance of CML stem/progenitor cells [20, 21]. Therefore, both LSD1 and SIRT1 can target on the same non-histone protein to modulate its functions. In addition, SIRT1 and LSD1 can co-exist within a repressor complex to regulate gene transcription [22]. However, it is unknown if LSD1 can regulate NHEJ and KU70 functions. We initially hypothesized that SIRT1 and LSD1 may co-regulate KU70 for NHEJ and mutation acquisition. Surprisingly, we discovered that SIRT1 and LSD1 compete for binding to KU70 in cancer cells in response to stress and have opposing roles in mediating NHEJ repair and mutation acquisition in CML and non-CML cells.

## RESULTS

### Opposing interaction with KU70 by LSD1 and SIRT1 in CML cells in response to stress and its impact on chromatin and DNA damage

**Figure 1:**
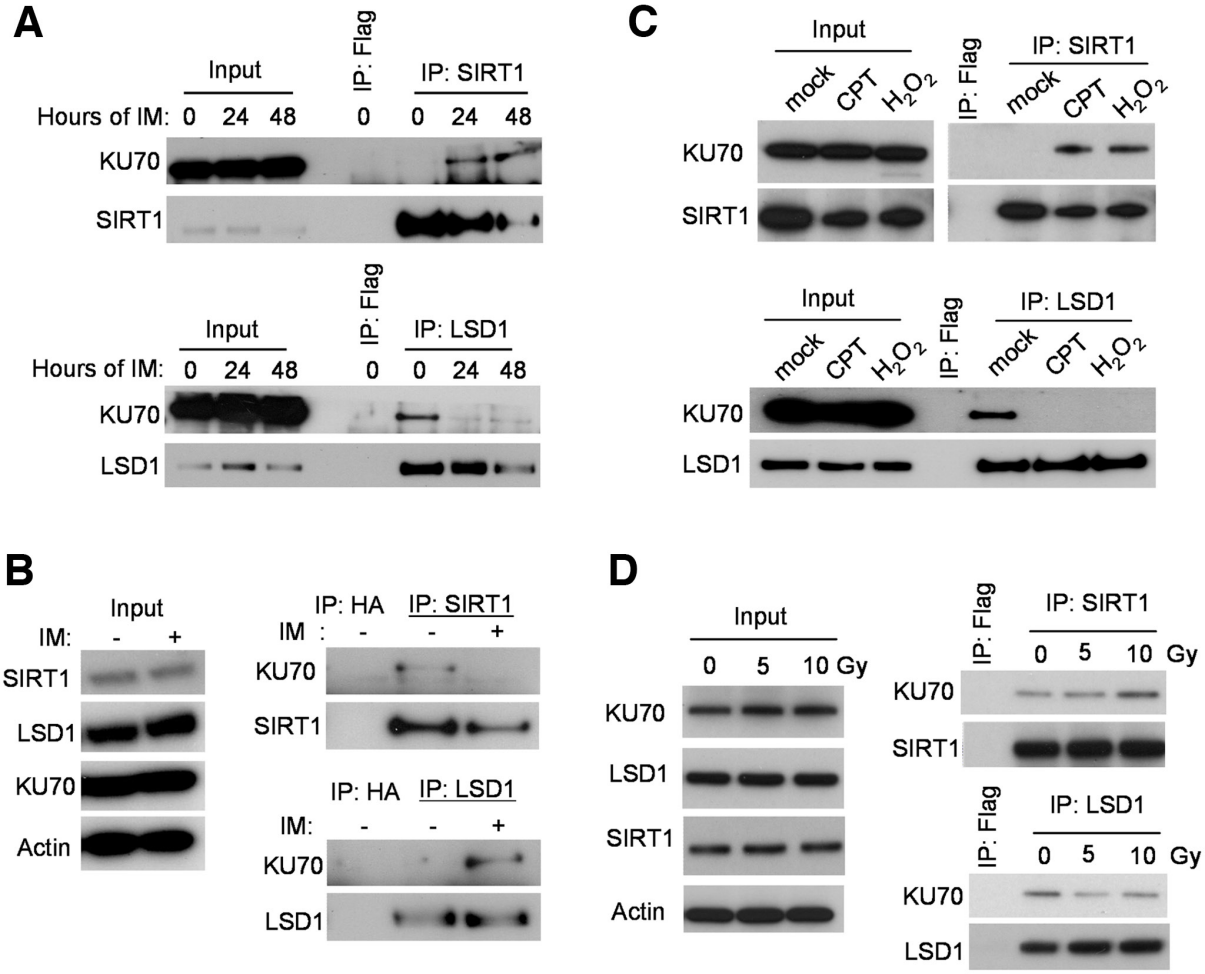
Stress-induced change of LSD1 and SIRT1 binding with KU70 **A.** Co-IP of endogenous KU70 with SIRT1 (upper panel) or LSD1 (lower panel) at baseline and two time-points (24 and 48 hours) following 2.5μM Imatinib (IM) administration in KCL-22 cells. **B.** Co-IP of KU70 with LSD1 or SIRT1 without and with IM treatment for 24 hours in KU812 cells. **C.** Co-IP of KU70 with LSD1 or SIRT1 in KCL-22 cells treated with 0.25 μM CPT for 12 h or 1 mM H_2_O_2_ for 1 h. **D.** Co-IP of KU70 with LSD1 or SIRT1 in KCL-22 cells after γ-irradiation. Cell lysates were prepared 2 hours after radiation.

Our initial co-immunoprecipitation (co-IP) pilot study indicated that both SIRT1 and LSD1 interacted with KU70. We set out to determine the potential roles of LSD1 and SIRT1 in regulation of KU70 in CML cell drug resistance. We used the KCL-22 cell model of CML acquired resistance to tyrosine kinase inhibitors that we previously developed [4]. By co-IP assay, we examined how SIRT1 and LSD1 may interact with KU70 in response to IM treatment. As shown in Figure 1A, KU70 interaction with SIRT1 was increased after IM treatment. Surprisingly, KU70 was bound to LSD1 in the untreated cells, but significantly dissociated from LSD1 after IM treatment. In contrast to KCL-22 cells, CML cell lines KU812 and K562 are unable to acquire BCR-ABL mutations for resistance [4]. Interestingly, opposite KU70 interaction pattern was observed in these cells, i.e. KU70 was bound to SIRT1 in the untreated KU812 (Figure 1B) and K562 (Supplementary Figure S1) cells, but after IM treatment, KU70 became more associated with LSD1. Similar to IM, increased interaction of KU70 with SIRT1 and reduced interaction with LSD1 occurred in KCL-22 cells treated with H_2_O_2_ and DNA damage agent camptothecin (CPT) (Figure 1C). Similar effects were also observed in KCL-22 cells after γ-irradiation, but to a lesser degree (Figure 1D). These results indicate that opposite SIRT1 and LSD1 interaction with KU70 may be a part of cellular stress response in cancer cells, especially for chemical-induced stress and damage.

**Figure 2:**
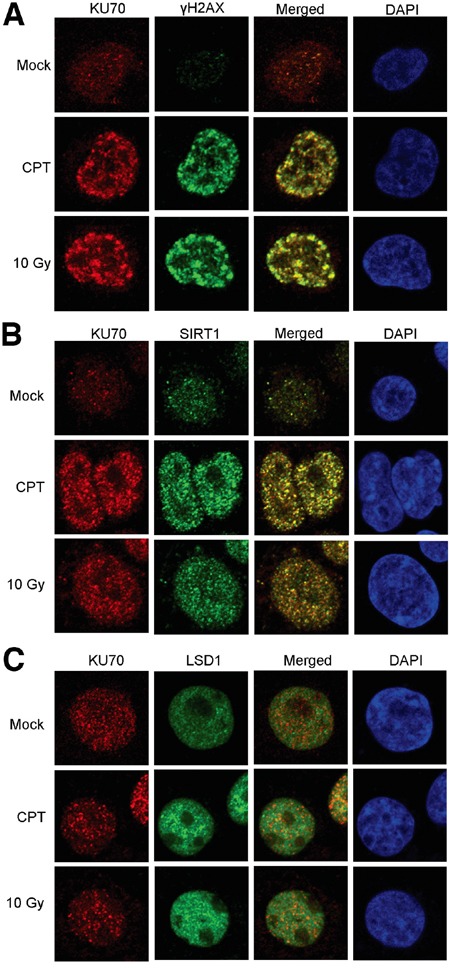
Confocal imaging of SIRT1, LSD1 and KU70 in KCL-22 cells after DNA damage Co-localization of KU70 with γH2AX **A.**, SIRT1 **B.** and LSD1 **C.** in the absence and presence of DNA damage by overnight treatment with 0.5 μM CPT or 2 h after 10 Gy radiation.

**Figure 3:**
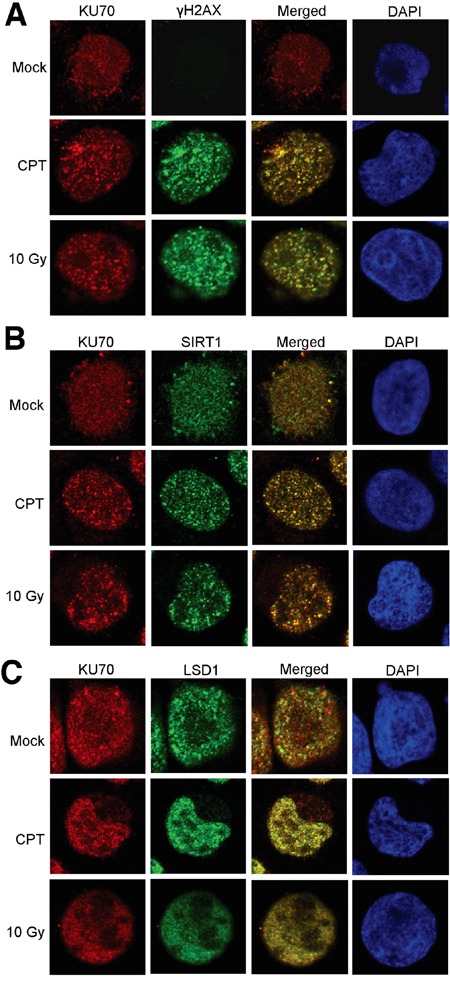
Confocal imaging of SIRT1, LSD1 and KU70 in KU812 cells after DNA damage Co-localization of KU70 with γH2AX **A.**, SIRT1 **B.** and LSD1 **C.** in the absence and presence of DNA damage by overnight treatment with 0.5 μM CPT or 2 h after 10 Gy radiation.

We next examined if altered KU70-SIRT1-LSD1 interaction could occur on DNA damage foci. By confocal imaging, we found that in KCL-22 cells KU70 largely aggregated on DNA damage foci marked by γH2AX after CPT treatment or γ-irradiation (Figure 2A). SIRT1 robustly co-localized with KU70 on these foci in CPT treated cells, and SIRT1 also co-localized with KU70 upon γ-irradiation but to a lesser degree than in CPT treated cells (Figure 2B). Although reduction of LSD1 interaction with KU70 was difficult to see in this assay, lack of co-localization of LSD1 with many KU70 foci upon damage was clearly evident (Figure 2C). These findings are consistent with co-IP results described above. In contrast, in non-resistant cell line KU812 cells, LSD1 co-localization with KU70 foci was substantially enhanced upon CPT treatment or γ-irradiation (Figure 3A, 3C). Intriguingly, recruitment of SIRT1 to KU70 foci also occurred after DNA damage, but it is difficult to judge whether there was quantitative reduction of its interaction with KU70 overall by imaging (Figure 3B). Nevertheless, the results suggest that differential KU70-SIRT1-LSD1 interaction occurs on DNA damage foci, particularly in the cells capable of acquiring resistant mutations.

**Figure 4:**
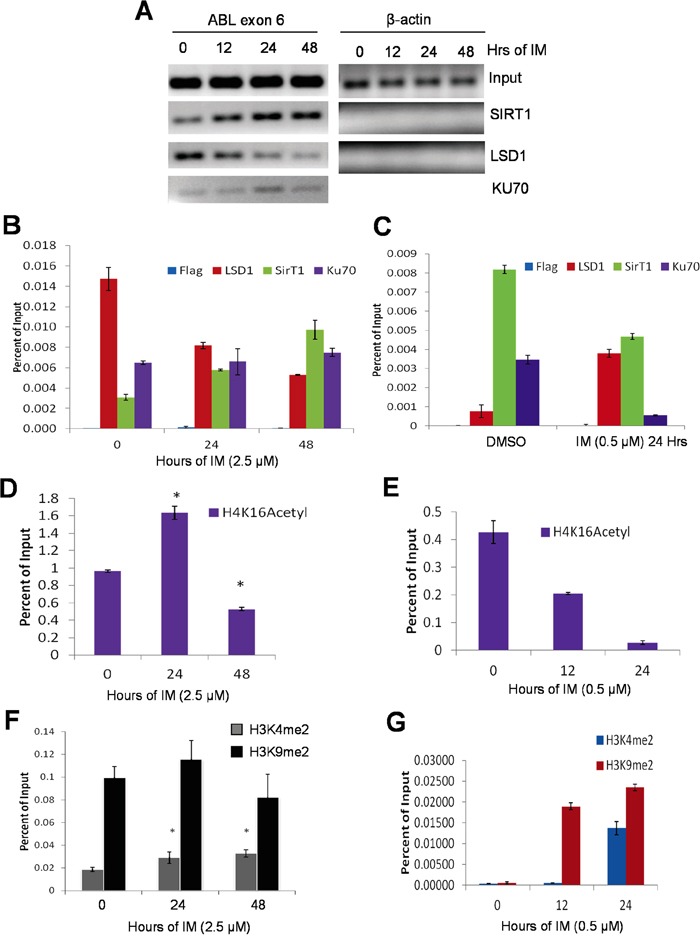
Opposite recruitment of LSD1 and SIRT1 to the ABL locus **A, B.** A, agarose gel images of standard ChIP-PCR for ABL exon 6 and β-actin in KCL-22 cells treated with 2.5μM IM for 0, 12, 24 or 48 hours. KU70 was not examined on β-actin. Primers that surround the β-actin transcription start site were used for PCR. B, quantitative ChIP-PCR of the ABL exon 6 of KCL-22 cells treated with 2.5μM IM for 0, 24 or 48 hours. Y-axis values are percents of Input for each respective time point. Bars are means of triplicates and error bars are standard error of the means (n=3). **C.** Bar graphs representing ChIP-qPCR of the ABL exon 6 of mock or 0.5μM IM treatment for 24 hours in KU812 cells; bars represent means and error bars are SEM. **D-G.** ChIP-PCR histone mark analysis of ABL exon 6 in KCL-22 cells treated with 2.5μM IM (D and F) IM and in KU812 cells treated with 0.5μM IM for indicated times. ChIP-PCR results were triplicates from a representative experiment, and similar patterns were observed in repeated experiments.

KCL-22 cells acquire T315I mutation of BCR-ABL for IM resistance [4]. We examined how KU70-SIRT1-LSD1 may be recruited on the ABL exon 6 where T315I mutation occurs. Using chromatin immunoprecipitation (ChIP) assay, we found that both LSD1 and SIRT1 occupied exon 6 of the ABL locus in untreated KCL-22 cells; upon IM treatment, the exon 6-bound LSD1 was reduced, but the abundance of SIRT1 was increased (Figure 4A, 4B). The levels of the exon 6-bound KU70 remained constant in untreated and IM treated cells. By contrast, in KU812 cells, the ABL exon 6-bound SIRT1 was reduced whereas LSD1 was increased following IM treatment (Figure 4C). The results on the ABL locus are consistent with that SIRT1 and LSD1 oppositely interact with KU70 globally in response to IM.

We carried out ChIP of active chromatin mark H4K16Ac in part as a positive control for KU70-SIRT1-LSD1 ChIP and to determine the impact of SIRT1 recruitment on local chromatin since BCR-ABL is actively transcribed in all CML cells. We found that H4K16Ac was comparably enriched on the ABL exon 6 in both KCL-22 and KU812 cells without IM treatment (Figure 4D, 4E), indicating active chromatin in these cells. Upon IM treatment, however, H4K16Ac was increased at 24 h and then decreased at 48 h, within 2-fold change in KCL-22 cells (Figure 4D), likely reflecting a chromatin dynamics seen during DNA repair [23, 24]. Surprisingly, H4K16Ac was substantially reduced for more than 10 fold by 24 h IM treatment in KU812 cells (Figure 4E) in spite of reduced recruitment of SIRT1 onto the locus. Therefore we further examined histone marks H3K4Me2 and H3K9Me2, both substrates of LSD1, on the ABL exon 6. Both dimethylation marks were readily detected in KCL-22 cells, and they changed only moderately with and without IM (Figure 4F). In contrast, both dimethylation marks were low in untreated KU812 cells, and were robustly increased upon IM treatment first by H3K9Me2 and then H3K4Me2 (Figure 4G). These indicate some baseline difference of the BCR-ABL locus chromatin and the response to IM in these cell lines. Sharp reduction of active mark H4K16Ac and surge of repressive mark H3K9Me2 may indicate formation of “repressive” chromatin structures upon DNA damage in KU812 cells. However, BCR-ABL mRNA levels did not change until a much later time (Supplementary Figure S2), suggesting that chromatin change may be related with DNA repair but not transcription.

**Figure 5:**
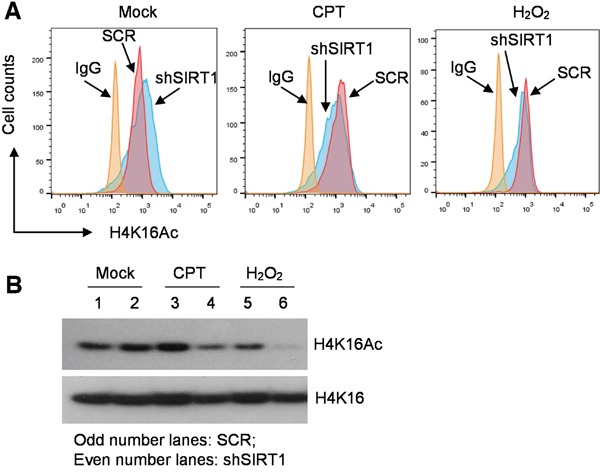
Distinct roles of SIRT1 in histone H4K16 acetylation in CML cells under normal or stressed conditions **A.** Flow cytometry analysis of H4K16Ac in KCL-22 cells with scrambled shRNA (SCR) or shSIRT1 knockdown, in the absence and presence of overnight treatment with 0.5 μM CPT or 200 μM H_2_O_2_. **B.** Western blot analysis of H4K16Ac of the cells shown in A.

The observed changes of chromatin marks are not quite consistent with classical functions of SIRT1 and LSD1 in histone modifications, especially for SIRT1 that is expected to deacetylate histone H4K16. In mouse embryonic stem cells and solid tumor cells, SIRT1 knockout or knockdown did not change global histone H4 acetylation [25, 26]; but upon DNA damage, SIRT1 can relocalize on chromatin and result in local changes of H4K16 acetylation and gene expression [12, 27]. However, how SIRT1 affects histone acetylation in hematological cells is not clear. We examined histone H4K16 acetylation in CML cells with SIRT1 gene knockdown in the absence and presence of DNA damage. By flow cytometry, we found that in the absence of DNA damage SIRT1 knockdown by shRNA increased global H4K16Ac in KCL-22 as expected; but in the presence of H_2_O_2_ or CPT, SIRT1 knockdown surprisingly reduced global H4K16Ac (Figure 5A). These results were confirmed by Western blot analysis (Figure 5B). Due to the difficulty in shRNA transduction of KU812 cells, we performed a similar study in IM-sensitive K562 cells. Likewise, we found that SIRT1 knockdown reduced H4K16Ac when the cells were under oxidative damage (Supplementary Figure S3). These findings suggest that SIRT1 may have distinct functions on histone modification under different conditions: reducing histone H4 acetylation under unstressed condition and increasing H4 acetylation under stressed condition. “Repressive” chromatin is considered unfavorable for DNA repair [28]. Therefore, SIRT1 recruitment to KU70 under stress may increase deacetylation of KU70 and possibly also modify other factors in the repair complex to maintain open chromatin for repair.

**Figure 6:**
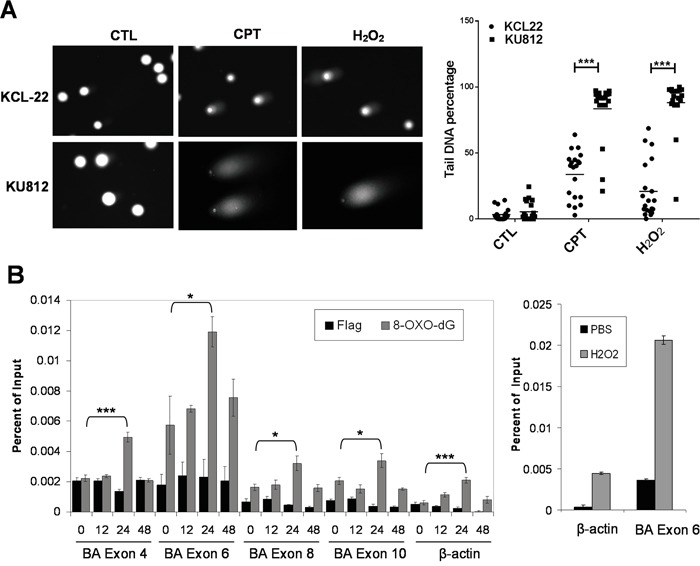
Global and locus-specific DNA damage in CML cells in response to stress **A.** Comet assay of KCL-22 and KU812 cells treated overnight with 0.5 μM CPT or 200 μM H_2_O_2_. CTL, control. Quantification of comet tail DNA was shown to the right. **B.** Left: real-time ChIP-PCR analysis of 8-Oxo-dG on ABL exons and β-actin after 2.5 μM IM treatment. Right: positive control of ChIP-PCR analysis of 8-Oxo-dG on ABL exon 6 and β-actin after 1 mM H_2_O_2_ treatment for 1 hour. * p<0.05; *** p<0.001.

Because differential KU70-SIRT1-LSD1 interaction is likely not restricted to one chromatin locus as indicated by co-IP assay, we hypothesized that the “repressive” chromatin orchestrated by KU70-SIRT1-LSD1 interaction, if extended beyond the ABL locus, could reduce overall DNA repair and result in more DNA damage in KU812 cells. We tested this by the comet assay of KCL-22 and KU812 cells. Indeed, KU812 sustained substantially more DNA damage than KCL-22 cells in response to the same DNA damage treatment (Figure 6A). Consistently, KU812 cells had much higher cell death rate under DNA damage than KCL-22 cells (not shown).

T315I mutation on the ABL exon 6 is exclusively acquired in KCL-22 cells in response to IM, and the BCR-ABL translocation locus has higher mutagenesis potential than other chromosomal loci [4]. To determine if the exon 6 has any difference from other ABL exons to DNA damage, we analyzed oxidative DNA damage by ChIP of 8-oxodeoxyguanine (8-Oxo-dG) in KCL-22 cells in response to IM. We found that the exon 6 had the highest spontaneous 8-Oxo-dG level among four ABL exons (4, 6, 8 and 10) analyzed, and also had the highest 8-Oxo-dG levels upon IM treatment, supporting its unique position for acquiring T315I mutation (Figure 6B). Spontaneous 8-Oxo-dG level on the exon 6 was also much higher than on the β-actin locus. As a positive control, we treated the cells with H_2_O_2_, and found that 8-Oxo-dG was proportionally increased on both β-actin locus and ABL exon 6, but the latter was subjected to much higher net increase of 8-Oxo-dG (Figure 6B, right panel). Interestingly, 8-Oxo-dG increase after IM treatment peaked at 24 h and then declined. This peak of oxidative DNA damage is in line with our previous observation that full block of BCR-ABL mutation acquisition by ATRA requires administration of ATRA not later than 24 h after initiation of IM treatment [6]. The ABL exon 4 was subjected to the second highest level of IM-induced oxidative damage (Figure 6B). Consistently, SIRT1 and LSD1 recruitment to the exon 4 was similarly altered to a certain degree (Supplementary Figure S4), indicating its potential for mutagenesis. In line with this, acquisition of exon 4 mutations E255K and Y253H can occur in subclones of KCL-22 cells [4], and as detailed below, robust knockdown of LSD1 could also trigger mutations on the exon 4.

### LSD1 and SIRT1 oppositely regulate NHEJ repair and BCR-ABL mutation acquisition

**Figure 7:**
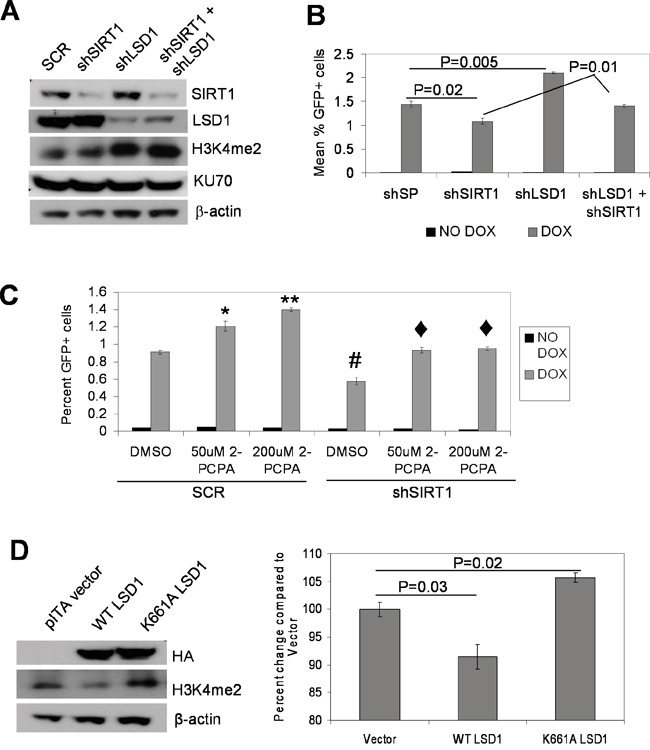
Opposing functions of LSD1 and SIRT1 in NHEJ repair **A.** Western blots of individual and combinatory knockdown of LSD1 and SIRT1. SCR, scrambled shRNA. **B.** NHEJ repair assay comparing individual and combinatory LSD1 and SIRT1 knockdown in KCL-22 cell-based NHEJ reporter cells. Bars represented mean percentage GFP positive cells with scrambled shRNA (shSP) or individual and combinatory gene knockdown. **C.** NHEJ reporter cells with SIRT1 or Scrambled (SCR) knockdown in the presence and absence of 50 or 200μM of the LSD1 inhibitor 2-PCPA. Dark gray bars represent percent of GFP cells without DOX induction, and light gray bars represent that with DOX induction. *P<0.01, **P<0.0001 and #P<0.01 when compared with SCR-DMSO; ♦P<0.01 compared with shSIRT1-DMSO. **D.** Left panel: immunoblots of NHEJ reporter cells transduced with vector alone, WT or K661A catalytically-inactive LSD1 expression vectors. HA-tagged LSD1 is detected using anti HA antibody. Right panel: comparing NHEJ repair with WT or K661A LSD1 over-expression. Bar graphs represented means (n=3) of percent GFP positive cells.

Next, we examined if interplay of SIRT1-LSD1-KU70 may actually affect KU70-mediated NHEJ repair. We previously showed that SIRT1 inhibition blocks BCR-ABL mutation acquisition, which is associated with reduction of KU70-mediated NHEJ repair [5]. Using the KCL-22 based NHEJ reporter cell line we engineered [5], we knocked down LSD1 and SIRT1 by shRNA individually and in combination. LSD1 and SIRT1 knockdown did not affect each other's gene expression, but LSD1 knockdown increased histone H3K4Me2 as expected (Figure 7A and Supplementary Figure S5). LSD1 knockdown enhanced, whereas SIRT1 knockdown reduced, NHEJ repair efficiency; double knockdown negated each individual's effect and returned the repair efficiency to the level of the scrambled shRNA control (Figure 7B). Likewise, treatment of NHEJ reporter cells with a LSD1 inhibitor, 2-Phenylcyclopropylamine (2-PCPA), enhanced repair efficiency in a dose dependent manner (Figure 7C). Furthermore, 2-PCPA treatment of SIRT1 knockdown reporter cells was able to return the repair efficiency to untreated scrambled control cells (Figure 7C). Consistently, lentiviral expression of WT LSD1 reduced NHEJ repair efficiency, while expression of demethylase-defective K661A LSD1 increased NHEJ efficiency (Figure 7D). These results show that SIRT1 and LSD1 oppositely regulate NHEJ repair, and lysine demethylase activity of LSD1 is required for such an action.

**Figure 8:**
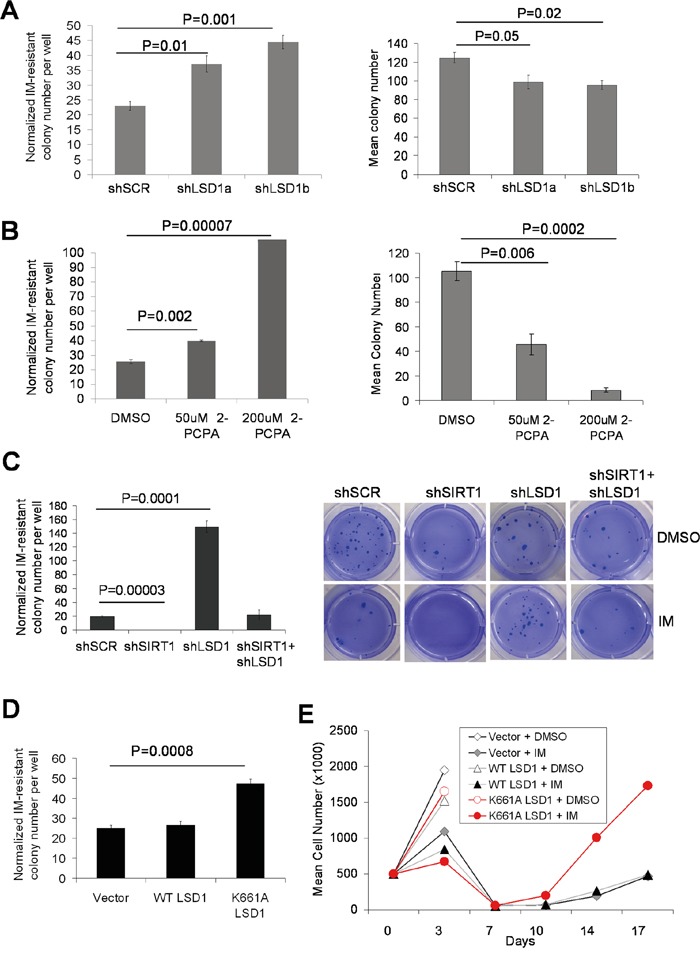
Opposing actions of LSD1 and SIRT1 in acquisition of BCR-ABL mutations **A-D.** Bar graphs representing relative mutation frequencies of imatinib-resistant KCL-22 colonies with 2.5μM IM treatment after normalization to DMSO mock treatment (plating efficiency). Values are means and error bars are SEM. A, left panel: change of mutation frequencies after LSD1 knockdown using two independent LSD1 shRNAs compared to Scrambled shRNA; right panel: plating efficiency. B, left panel: effect of LSD1 inhibitor 2-PCPA on KCL-22 cell mutation upon IM treatment; right panel: plating efficiency. Treatment of 2-PCPA was started 72 hours before IM treatment was initiated to simulate LSD1 knockdown. C, left panel: relative mutation frequencies of KCL-22 cells after knockdown of LSD1, SIRT1, double knockdown of LSD1/SIRT1 or Scrambled shRNA; right panel: representative images of IM-resistant cell colonies. D, relative mutation frequencies of KCL-22 cells stably expressing WT LSD1, catalytically-inactive K661A LSD1 or vector alone. **E.** Effect of mutant LSD1 expression on KCL-22 cell relapse on IM in liquid culture. Growth curves showing cell counts of KCL-22 cells stably expressing WT LSD1, K661A LSD1 or vector alone, in the presence and absence of 2.5μM IM.

We next examined the roles of LSD1 in BCR-ABL mutation acquisition and IM resistance in CML cells. Knockdown of LSD1 in KCL-22 cells only moderately reduced cell proliferation, increased G2/M arrest and increased apoptosis (Supplementary Figure S6). More significant growth inhibition and apoptosis induction after LSD1 knockdown or treatment with 2-PCPA were observed in acute myeloid leukemia cell line TF-1 (Supplementary Figure S7), consistent with previous findings [29, 30]. Despite reduced colony formation of KCL-22 cells on soft agar after LSD1 knockdown (i.e. the plating efficiency), we found that IM-resistant mutant colony formation as a result of BCR-ABL mutations in LSD1 knockdown KCL-22 cells did not reduce or even increased, and when normalized to the plating efficiency, the relative mutant colony formation was significantly increased (Figure 8A and Supplementary Figure S8A). Similar results were seen with chemical inhibition of LSD1 by 2-PCPA (Figure 8B and Supplementary Figure S8B). In contrast to LSD1 knockdown, SIRT1 knockdown completely blocked IM-resistant colony formation, and the combination of SIRT1 and LSD1 knockdown neutralized each other's effect (Figure 8C), similar to that for NHEJ repair described above.

We further examined the effect of over-expression of WT and catalytically inactive K661A LSD1 in KCL-22 cells. Cell apoptosis analyzed by annexin V labeling was not affected by either WT or K661A LSD1 expression in the presence or absence of IM treatment (data not shown). Ectopic expression of K661A LSD1 increased BCR-ABL mutation frequency (Figure 8D), and K661A LSD1 expressing KCL-22 cells showed quicker relapse in liquid culture (Figure 8E); however, we did not observe such an effect of WT LSD1 over-expression. The lack of effect of WT LSD1 expression is possibly due to abundant presence of LSD1 on the ABL locus (Figure 4A, 4B) that would make it more difficult to have an impact by ectopic WT LSD1, whereas K661A LSD1 may have a dominant negative effect.

To determine if resistant clones after LSD1 knockdown or 2-PCPA treatment also acquire genetic mutations, we sequenced BCR-ABL exons. Indeed nearly all resistant clones acquired BCR-ABL mutations (Supplementary Figure S9A). Interestingly, whereas most resistant clones had T315I mutation on the exon 6 as expected, clones from one set of knockdown (shLSD1b) mostly acquired Y253H mutation on the exon 4. Although the precise reason why shLSD1b yielded different mutation spectrum is unclear, we noticed shLSD1b tended to yield more efficient LSD1 knockdown in KCL- 22 cells (Supplementary Figure S9B). This suggests that a threshold level of LSD1 might be needed to maintain chromatin functions and mutagenesis on the exon 6 as described above. In line with this note, one resistant clone from a higher concentration of 2-PCPA treatment did not acquire T315I mutation (Supplementary Figure S9A).

### LSD1 and SIRT1 oppositely regulate HPRT mutation acquisition in prostate cancer cells

**Figure 9:**
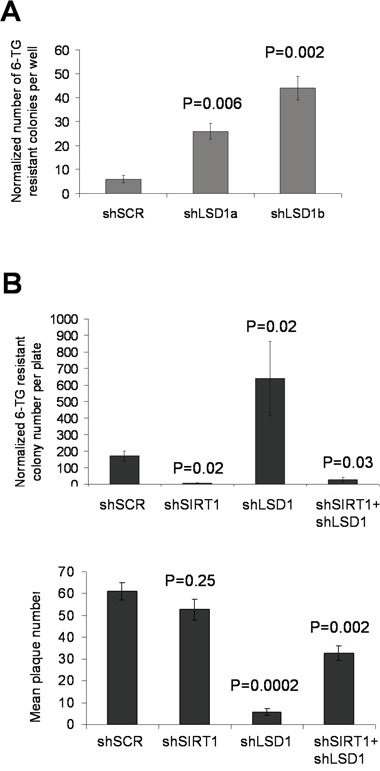
Opposing actions of LSD1 and SIRT1 in acquisition of HPRT mutations **A.** Spontaneous HPRT mutations in KCL-22 cells following LSD1 knockdown. 6-TG resistant colonies per million cells per well were scored, and normalized to the plating efficiency that was measured by soft agar colony numbers in the absence of 6-TG. **B.** Upper panel: HPRT mutations in prostate cancer PC3 cells upon individual and combinatory SIRT1/LSD1 knockdown. DNA damage was induced by 0.5μM camptothecin (CPT) treatment for 1 hour, and 15 million cells per plate were seeded for 6-TG selection. Plaques were scored and normalized to the plating efficiency (plaques in the absence of 6-TG) shown in the lower panel. P values were indicated as comparison to shSCR control in both A and B.

We next determined the roles of LSD1 and SIRT1 in regulating mutation acquisition in other genes and cell types. We first performed hypoxanthine-guanine phosphoribosyl-transferase (HPRT) mutation assays in KCL-22 cells with LSD1 knockdown. We found that spontaneous HPRT mutation frequency in KCL-22 measured by 6-thioguanine (6-TG) resistant colony formation was significantly increased with LSD1 shRNAs compared to scrambled control (Figure 9A), an effect opposite to SIRT1 knockdown we showed before [5]. We then examined HPRT mutations in prostate cancer PC3 cells with LSD1, SIRT1, combined LSD1/SIRT1 or scrambled shRNA knockdown. Since PC3 cells have a relatively low spontaneous mutation rate [5], we treated these cells with CPT to induce DNA damage. LSD1 knockdown significantly increased formation of 6-TG resistant plaques in spite of a reduced plating efficiency, while SIRT1 knockdown largely abolished resistant plaque formation without significantly affecting the plating efficiency; combined LSD1/SIRT1 knockdown partially restored 6-TG resistant plaque numbers relative to SIRT1 knockdown alone, 27.4±13.4 vs 4.2±3.7, respectively (mean±SEM; Figure 9B and Supplementary Figure S10). Together, these data suggest that LSD1 and SIRT1 oppositely regulate cellular NHEJ repair and acquisition of genetic mutations in cancer cells in response to DNA damage or therapeutic stress, a phenotype consistent with their opposing interaction with KU70.

### LSD1 and SIRT1 compete for interaction with KU70

**Figure 10:**
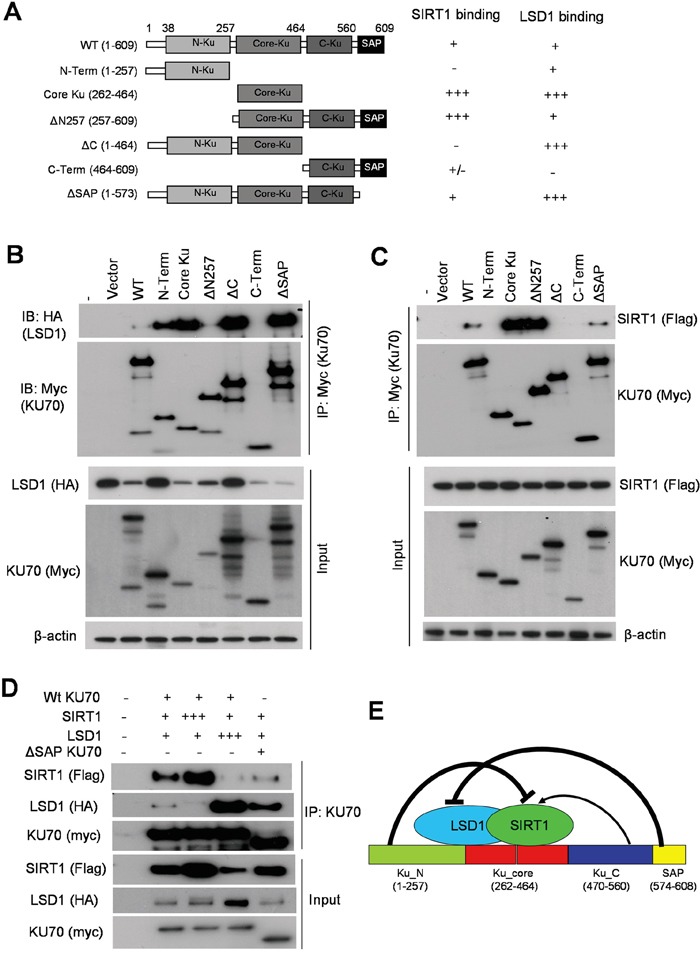
Competitive binding of KU70 by LSD1 and SIRT1 **A.** Summary of domain mapping of KU70 interaction with LSD1 and SIRT1 in HEK293T cells. Left panel depicts myc-tagged KU70 constructs used along with either wild-type HA-tagged LSD1 (in B) or flag-tagged SIRT1 (in C). Table on the right summarizes relative binding strength for KU70 domains with LSD1 or SIRT1. **B, C.** Co-IP of myc-KU70 followed by immunoblots of HA-tagged LSD1 (B) or flag-tagged SIRT1 (C) with co-transfected myc-KU70 from HEK293T cells. **D.** Co-IP of WT or ΔSAP myc-tagged KU70 with varying amounts of WT-SIRT1, WT-LSD1 or vector alone in HEK293T cells. Total amount of transfected DNA was held constant by using empty vector DNA to equalize all samples. **E.** Schematic summary of KU70 domains for regulating interaction with LSD1 and SIRT1. Both SIRT1 and LSD1 bind to the KU70 core domain. KU70 N-terminus strongly represses SIRT1 binding but C-terminal linker region facilitates SIRT1 interaction. KU70 C-terminal SAP motif strongly represses LSD1 binding.

To determine the mechanisms how SIRT1 and LSD1 oppositely interact with KU70 and regulate its functions, we mapped the domains within KU70 that are responsible for interaction with LSD1 and SIRT1, respectively. We engineered several Myc-tagged human KU70 constructs (Figure 10A) and co-expressed each with HA-tagged WT LSD1 in 293T cells. Co-IP assays with anti-Myc resin showed that LSD1 bound robustly to the core domain (262-464) of KU70 and partially to the N-terminus, and KU70 lacking the N-terminal domain (ΔN257) only weakly interacted with LSD1 (Figure 10A, 10B). Interestingly, deletion of the SAP domain (574-609) at the C-terminus of KU70, a domain that may help KU70-DNA interaction [31] and mediate BAX protein interaction for cell survival [32], dramatically increased KU70 interaction with LSD1, indicating that SAP was a strong repressor motif for LSD1 interaction (Figure 10A, 10B). An unexpected variation of input HA-LSD1 expression was noticed when it was co-transfected with KU70 constructs (Figure 10B). This was reproduced in independent mappings, but it did not affect the conclusions of KU70/LSD1 interaction domains (Supplementary Figure S11).

Co-IP assays of Myc-tagged KU70 revealed that the core domain (262-464) of KU70 primarily mediated the interaction with flag-tagged SIRT1. The KU70 N-terminus did not bind SIRT1 but acted as a strong repressor for SIRT1 binding to the core (Figure 10A, 10C). SIRT1 only very weakly interacted with the C-terminal regions of KU70, but the sequence (464-573) between the core and SAP domains, which harbors the nuclear localization signal (NLS) and linker, was a positive regulatory domain for SIRT1 interaction and antagonized the repression from the N-terminus (Figure 10A, 10C). Noticeably, the NLS/linker region has multiple sites for lysine acetylation, two of which are targeted by SIRT1 [11, 13].

Given that the KU70 core mediated strong interaction with both SIRT1 and LSD1, we hypothesized that LSD1 and SIRT1 may competitively bind to KU70. To test this, we titrated the amount of either flagged-SIRT1 or HA-LSD1 for transfection aiming to compete out the binding of the other to Myc-KU70. After co-expressing three constructs in differing molar ratios in 293T cells, we found that the more SIRT1 was present, the less relative LSD1 bound to KU70, and vice versa (Figure 10D). Consistently, under equal molar ratio ΔSAP KU70 had stronger interaction with LSD1 than WT KU70, and conversely WT KU70 had stronger interaction with SIRT1 than ΔSAP KU70. These results suggest a competition of LSD1 and SIRT1 for binding to KU70 as summarized in Figure 10E.

### LSD1 and SIRT1 competitive interaction with KU70 regulates BCR-ABL mutation acquisition

**Figure 11:**
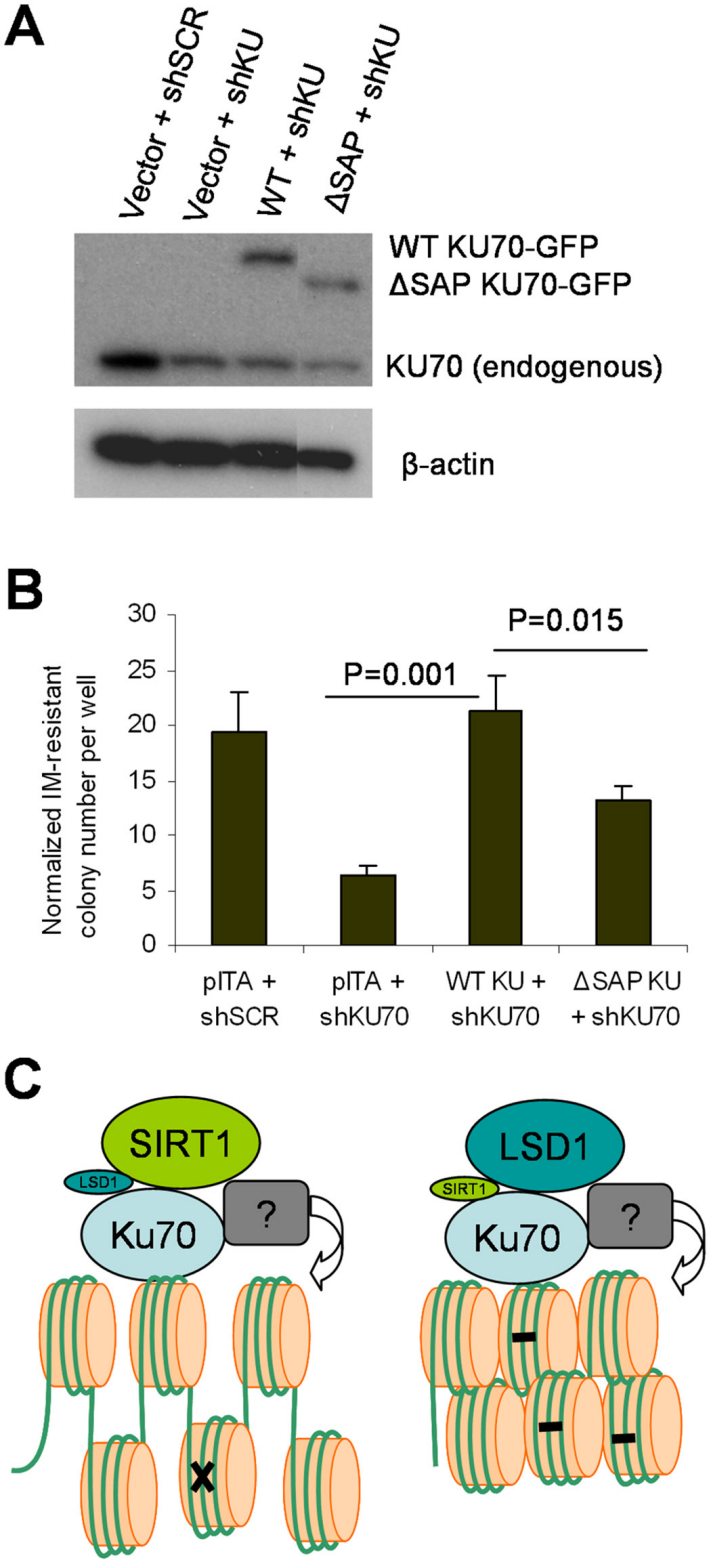
The KU70 regulatory domain for SIRT1/LSD1 competition regulated BCR-ABL mutation **A.** Western blot analysis of expression of EGFP-fusion WT or ΔSAP KU70 in KCL-22 cells with knockdown of endogenous KU70 by shRNA targeting 3′ UTR. The ΔSAP KU70 lane was spliced adjacent to the rest of the blot from the same gel. **B.** The effect of EGFR-fusion constructs on restoring imatinib-resistant BCR-ABL mutant colony formation in KCL-22 cells with KU70 3′ UTR knockdown. **C.** A schema showing roles of SIRT1 and LSD1 in regulating KU70 for DNA repair. With enhanced SIRT1-KU70 interaction (left), repair is increased with more open chromatin but has more likelihood for acquiring mutation (indicated by x) and developing drug resistance. With enhanced LSD1-KU70 interaction (right), repair is reduced with more “repressive” chromatin resulting in accumulation of DNA damage (indicated by -) and cell lethality. SIRT1 and LSD1 may indirectly modify histones via unidentified factors(indicated by ?) associated with the repair complex.

To further determine if LSD1/SIRT1 competitive interaction with KU70 may indeed affect BCR-ABL mutation acquisition, we over-expressed WT or ΔSAP KU70 into CML cells. However, our multiple efforts to over-express KU70 in CML cells failed, possibly because KU70 is one of the most abundant proteins in human cells [33] or its expression is under tight control. To aid ectopic KU70 expression, we designed shRNA targeting KU70 3′ untranslated region (3′UTR) to reduce endogenous KU70 without affecting ectopic KU70 cDNA. In addition, to help visualize ectopic KU70 expression and transduction efficiency, we made EGFP (enhanced green fluorescent protein) fusion to KU70. EGFP-KU70 fusion preserves KU70 functions to be properly targeted to laser–induced DNA damage sites [34]. KCL-22 cells were transduced with lentiviral expression vectors for EGFP-KU70 (WT or ΔSAP mutant), enriched by puromycin selection, and followed by KU70 3′UTR shRNA knockdown. Using this strategy, EGFP-KU70 (WT and mutant) were expressed to the levels about same as that of the remaining endogenous KU70 (Figure 11A). We found that KU70 3′UTR knockdown reduced BCR-ABL mutant formation, which was completely rescued by WT KU70 expression; in contrast, ΔSAP KU70 mutant had significantly lower capability to restore BCR-ABL mutations (Figure 11B). These results are consistent with stronger LSD1 interaction with ΔSAP KU70 that reduces SIRT1 interaction and thus KU70 activation. All together, our data support that competitive interaction of KU70 by LSD1 and SIRT1 in cancer cells in response to stress regulates NHEJ repair and mutation acquisition.

## DISCUSSION

In this study, we identified a novel cellular stress signaling mechanism in which lysine deacetylase SIRT1 and demethylase LSD1 competitively binds to KU70 in response to chemotherapeutic agents and DNA damage. Such competitive KU70 binding affects a cancer cell's ability to repair broken DNA and acquire genetic mutations. Both SIRT1 and LSD1 strongly bind to the KU70 core domain, providing a molecular basis for such competition. These findings shed new insight of CML acquired resistance and may help better understand mutation acquisition process under stress conditions and genetic evolution of cancer.

We showed that differential recruitment of SIRT1 and LSD1 to KU70 occurred on DNA damage foci globally, as well as locus-specifically on the ABL exon 6 where the BCR-ABL mutation (T315I) hot spot is located. Although SIRT1 and LSD1 are known histone modifiers, their recruitment to the chromatin locus in response to stress did not correlate well with the expected histone mark changes, suggesting their main roles under stress are not to directly modify histones. This is particularly evident that SIRT1 knockdown increased H4K16Ac under non-stressed condition, but reduced H4K16Ac under stress. In terms of chromatin, the recruitment of SIRT1 to DNA damage foci possibly would modify other factors to maintain open chromatin for DNA repair. This can be further coupled with SIRT1 deacetylation of KU70 to enhance repair. However, the enhanced repair is ostensibly associated with generation of rare genetic mutations for drug resistance. In contrast, increased recruitment of LSD1 at the expense of SIRT1 to DNA damage foci would lead to “repressive” chromatin that is not favorable for repair, resulting in accumulation of DNA damage (Figure 11C), and cells with sustained DNA damage would die. However, knockdown of LSD1 alone was insufficient to convert IM-sensitive cell lines to acquire BCR-ABL mutations for resistance (data not shown), suggesting that additional chromatin regulatory factors are involved in the process.

Both SIRT1 and LSD1 target lysines for protein modification, such as in the case of p53 [19, 35]. It is tempting to speculate that LSD1 may demethylate some same lysine residues on KU70 as SIRT1 deacetylates for their competitive regulation of KU70 functions. A recent proteomic study identifying lysine methylation of KU70 and several other DNA repair factors [36] further suggests such a possibility. However, whether KU70 is a methylated protein remains to be validated and whether LSD1 can demethylate KU70 for regulating NHEJ warrants further investigation. As mentioned above, it is also possible that LSD1 may target factors other than KU70 within the NHEJ repair complex. In this aspect, LSD1/SIRT1 competitive interaction with KU70 would still serve as a mechanism to modulate SIRT1 activation of KU70 functions. Regardless, our findings raise a possibility that KU70-mediated NHEJ is subjected to dynamic epigenetic regulation in response to cellular stress. In addition, we speculate that LSD1/SIRT1 competitive interaction with KU70 may have an impact on other KU70 functions beyond DNA repair, such as certain gene expression and cell survival.

In response to IM treatment, increased binding of KU70 to SIRT1 and reduced binding to LSD1 occur on the ABL locus in relapse-prone cells. Such switching correlates with enhanced DNA damage repair and acquisition of T315I mutation on the locus in these cells whereas the opposite change occurs in CML cell lines unable to acquire resistant mutations. Interestingly, spontaneous oxidative DNA damage was detected on the ABL exon 6, and SIRT1, LSD1 and KU70 were found on the locus even in the absence of TKI treatment stress. Exon 6 is also subjected to the highest level of oxidative DNA damage upon IM stress. These findings explain why the exon 6 is a hot spot for T315I mutation and lend support to our previous prediction that the endogenous BCR-ABL locus may have ongoing spontaneous DNA damage and repair under the steady state, which may be perturbed by environmental stress, accentuating mutagenesis processes [37]. SIRT1 can be recruited to sites of DNA damage [12, 27] and Mosammaparast et al showed that LSD1 is recruited to DNA damage foci in the S/G2 phase [38]. It is possible that spontaneous DNA damage on the BCR-ABL locus triggers recruitment of LSD1, SIRT1 and KU70, although the source of such damage remains to be determined. Imatinib treatment of CML cells quickly causes cell cycle arrest at G0/G1 [39] where NHEJ operates predominantly [40], but the treatment stress elicits a new wave of modification of DNA repair in arrested cells by altering LSD1 and SIRT1 interaction with KU70 and likely changing repair kinetics and fidelity, which has crucial impact on mutation acquisition. However, detailed signaling mechanisms for competitive KU70 binding by LSD1 and SIRT1 remain to be determined.

Although both SIRT1 and LSD1 inhibition leads to suppression of cell growth and soft agar colony formation, the mutation frequency is increased with LSD1 inhibition but reduced with SIRT1 inhibition. This difference is also evident on actual mutant colony formation even without normalization to the plating efficiency, regardless of that the knockdown of these genes has more or less impact on the plating efficiency. The change of BCR-ABL mutation frequency upon SIRT1 or LSD1 alteration is correlated with the change of NHEJ repair efficiency. However, it is currently unclear how NHEJ is molecularly associated with acquisition of point mutations of BCR-ABL, given that NHEJ is generally believed to produce insertions and deletions. Interestingly, Jan Vijg and colleagues showed that LacZ reporter mice deficient in NHEJ with Ku80 knockout have significantly reduced point mutations in liver and brain and moderately reduced point mutations in spleen, and importantly these point mutations contain largely base substitutions [41]. Reduction of point mutations in liver and brain was also reported by a different group using the same mice [42]. Although the mechanisms for reduction of those point mutations with defective NHEJ in vivo are also unknown, their findings are consistent with our finding of reduced BCR-ABL mutations upon inhibiting NHEJ in the KCL-22 CML cell model. Furthermore, Jan Vijg and colleagues showed that acute H_2_O_2_ treatment of mouse embryonic fibroblasts derived from the Ku80^−/−^ mice do not lead to a significant change of mutations as compared to Ku80^+/+^ fibroblasts, and suggested that in vivo accumulation of mutations over time may account for the difference of mutation frequencies in mouse organs [41]. In cancer cells, however, we speculate that much accelerated genome evolution with altered DNA repair systems would occur, and this in turn allows rapid detection of change of mutation rate upon NHEJ inhibition in cell culture. With that said, however, we did not find point mutations by Sanger sequencing of EJ5 NHEJ reporter products in CML cells (data not shown). This may be due to the limited number of clones (<100) we analyzed, but it also remains possible that changes of KU70/80 may impact on other repair pathway functions to indirectly affect point mutations.

Although blockade of LSD1 inhibits tumor growth and induces apoptosis, LSD1 inhibition enhances the cancer cell's NHEJ repair activity, which may contribute to acquisition of resistant mutations. In this regard, it is known that NHEJ pathway is aberrantly activated in myeloid leukemia that contributes to chromosomal rearrangements and chemoresistance [43, 44]. Besides, LSD1 inhibition promotes hematopoietic stem/progenitor cell expansion and prevents differentiation and maturation [45, 46]. Our results along with those of others may imply potential unintended consequences of anti LSD1 therapies for certain neoplastic diseases. Further study in patients will be needed to corroborate this. Nevertheless, our study sheds novel insight of SIRT1/LSD1/KU70 signaling and functions in cancer cells under therapeutic and environmental stress, and helps understand mechanisms of cancer drug resistance.

## MATERIALS AND METHODS

### Cell lines, small molecule inhibitors and plasmids

The chronic myelogenous leukemia cell lines KCL-22, K562 and KU812 cells were grown in RPMI-1640 supplemented with 10% fetal bovine serum (FBS, Hyclone) with penicillin/streptomycin. TF-1, the acute myeloid leukemia cell line, was grown in RPMI-1640 with 10% FBS, 2ng/mL of human granulocyte-macrophage colony-stimulating factor (GM-CSF) and antibiotics. HEK293T cells were cultured in DMEM supplemented with 10% FBS and antibiotics. Imatinib (IM or STI, LC Labs) was dissolved in dimethyl sulfoxide (DMSO) and used at 0.5μM for K562 and KU812 cells or 2.5μM for KCL-22 cells. The irreversible LSD1 inhibitor, 2-Phenylcyclopropylamine (2-PCPA, Cayman Chemical) was dissolved in DMSO and used as indicated.

Human WT and K661A LSD1 cDNA was a generous gift from Dr. Yang Shi (Harvard Medical School). Complementary DNA was subcloned into pITA. puro lentiviral vector. The pITA. puro vector was derived from pHAGE.UBC. ZsGreen (Dr. Richard Mulligan, Harvard Medical School) with an EF-1 promoter replacing the UBC promoter. Human KU70 cDNA was generously provided by Shigemi Matsuyama (Case Western Reserve University, Cleveland, OH). For lentiviral expression of KU70, the cDNA was subcloned into pITA.puro. For lentiviral-based short hairpin RNA (shRNA) knockdown, oligonucleotide sequences were designed using the PSICOOLIGOMAKER program and cloned into pSICOR.puro. For lentiviral particle generation, HEK293T cells were co-transfected with pLP1, pLP2, VSV-G and lentiviral vector using the calcium phosphate method described previously [4]. Viral supernatants were collected 72 hours post transfection, filtered and concentrated using poly-ethylene glycol (Sigma).

### Co-immunoprecipitation (Co-IP) and western blot analyses

Cells were lysed in IP buffer with complete protease inhibitor cocktail (Roche). For Co-IP of exogenously expressed proteins, 150μg of total extracts were used for each IP reaction. For endogenous protein co-IP reactions, 1mg of total protein was used per reaction. Extracts were pre-cleared with 20μL protein A/G agarose beads (Santa Cruz Biotechnology) for 1 h. Pre-cleared lysates were incubated with primary antibody on a rotator overnight at 4°C. Protein A/G agarose beads were then added for 2 h. Agarose beads were washed 3 times in IP buffer and eluted with sample buffer after boiling for 5 min. Proteins were run on NuPAGE Novex 4-12% Bis-Tris gradient gels with MOPS buffer (Life Technologies). Antibodies used for IP and IB: LSD1 (Cell Signaling, cat#2184), SIRT1 (Abcam, cat# ab32441), KU70 (A/G conjugated agarose from Santa Cruz, cat# sc-1486AC and non-conjugated form from Thermo #N3H10), KU80 (Cell Signaling), mono and di methyl lysine (Abcam), H3K4me2 (Abcam), Flag (M2 from Sigma), HA (Sigma), Myc conjugated agarose (Sigma).

### Confocal imaging

KCL-22 and KU812 cells were treated with 0.5 μM CPT for 16h or 10 Gy γ-irradiation. The cells were harvested and about 3,000 cells were cytospun onto the slides. The cells were fixed by 2% paraformaldehyde for 15 min at room temperature, followed by permeabilization with 0.1% triton X-100 in 5% BSA for 30 min, and then stained with anti-phospho-γ-H2AX (Cell Signaling, #2577s), anti-SIRT1 (Cell Signaling, #8469s), anti-LSD1 (Cell Signaling, #2184S) and anti-KU70 (Santa Cruz, sc1486) at dilution of 1:200 at 4°C overnight. The Alexa Fluor 594 or Alexa Fluor 488 conjugated anti-mouse/anti-rabbit/anti-goat secondary antibodies (Themo Scientific) were used at a dilution of 1:500 for 1 h on ice. After washing for 3 times, the slides were mounted with an anti-fade reagent with DAPI (Themo Scientific, S36938). Confocal imaging was performed with a Zeiss LSM 700 Confocal Microscope, and images were taken with a 20X objective.

### Comet assay

The assay was performed with the Comet Assay Kit (TREVIGEN #4250-050-K) per the manufacturer's instructions. Briefly, the cells were harvested and mixed with molten LM Agarose at 1×10^5^/mL at 37°C at ratio of 1:10 (v/v), and 50 μL mixture was immediately added onto CometSlide. After gelling, the slides were immersed in 4°C lysis solution for 60 min and then incubated in alkaline unwinding solution for 20 min. Then the slides were placed in electrophoresis tray with 4°C alkaline electrophoresis solution and run 30 min at 25 V. The slides were gently rinsed with water and incubated in 70% ethanol for 5 min. The slides were dried at 37°C and then stained with 100 μL diluted SYBR Gold solution for 30 min in dark. After rinse briefly in water, the slides were dried completely at 37°C and ready for imaging by fluorescent microscopy. The images were analyzed by Comet Assay software (Perceptive Instruments) and the percentage of tail DNA was measured to evaluate the DNA damage.

### Flow cytometry analysis of histone H4K16 acetylation

Cells were treated with CPT or H_2_O_2_ as indicated. The cells were harvested, spun down and washed with phosphate-buffered saline (PBS). The cells were stained with DAPI for 5 min and washed with PBS. Then the cells were fixed with 2% paraformaldehyde in PBS for 20 min at room temperature, followed by washing with PBS. The cells were then permeabilized with PBS supplied with 0.5% triton X-100 and 5% BSA. After that the cells were incubated with anti-acetylated H4K16 antibody (Millipore, #07-329) at dilution of 1:100 overnight at 4°C on a shaker. After wash with PBS for 3 times, the cells were stained with a second fluorescent antibody (Alexa Fluor 594) at dilution of 1:1000 for 1 hour. The cells were washed 3 times and analyzed by flow cytometry.

### Chromatin immunoprecipitation (ChIP) assay

ChIP was performed as previously described [47]. Briefly, 1×10^7^ CML cells were cross-linked with 1% formaldehyde for 15 min. Reactions were stopped with 125mM Glycine. Nuclear extracts were sheared by sonication and diluted 1:10 in IP buffer for immunoprecipitation. Protein A/G beads (Santa Cruz Biotechnology) were washed and eluted with 1% SDS followed by reverse cross-linking and proteinase K digestion. ChIP DNA was purified using phenol/chloroform extraction method. For PCR amplification of ChIP DNA, Taq Red PCR mix (Sigma) was used for 30 cycles and PCR products were run on a 1.5% agarose gel. For quantitative real-time PCR (qPCR), SYBR green master mix (KAPA Biosystems) was used and run in a BioRad iCycler. The following antibodies were used for ChIP: LSD1 (Cell Signaling, cat#2184), SIRT1 (Abcam, cat# ab32441), Ku70 (Thermo #N3H10), histone H4 K16Ac (Millipore, cat#07-329), histone H3 K4Me2 (Abcam, cat# ab32356), histone H3 K9Me2 (Abcam, cat#ab32521) and, 8-Oxo-deoxyGuanine (Trevigen, cat# 4354-MC-050).

### Mutation frequency, relapse and HPRT assays

These assays were performed as previously described [4, 5]. Briefly, for IM resistance, 1×10^6^ KCL-22 cells were plated in soft agar with 2.5μM IM in a 6-well plate. Separately, 500 KCL-22 cells were seeded in soft agar without IM and the colony formation rate was referred to as the plating efficiency. At 21 days post seeding, cells are stained with crystal violet and counted. The mutation frequency is calculated as the number of TKI resistant colonies in the IM treated relative to the plating efficiency for each experimental condition. Another assay to measure acquired TKI resistance in KCL-22 cells is by measuring cell proliferation in liquid culture with 2.5 μM IM and counting viable cells using trypan blue exclusion every 3 days. For this assay, 5×10^5^ KCL-22 cells were seeded in a 1mL of culture medium with IM in a 24 well plate.

For Hypoxanthine-guanine phosphori-bosyltransferase (HPRT) mutation assays, KCL-22 and PC3 cells were treated with HAT (Hypoxanthine, Aminopterin, Thymidine; Sigma) containing RPMI-1640 medium followed by recovery with HT medium without aminopterin to screen out cells that contain pre-existing HPRT mutations. [4] For KCL-22 cells, no mutagenesis induction was necessary, whereas PC3 cells were treated with 0.5μM camptothecin (CPT, Sigma) for 1 h to induce mutagenesis. To quantify spontaneous mutation frequency in KCL-22 cells, 1×10^6^ cells were seeded with 40μM 6-thioguanine 6-thioguanine (6-TG) in soft agar on a 6-well plate. For plating efficiency, 500 cells were seeded in soft agar without 6-TG selection. For PC3 cells, 1.5×10^7^ cells were seeded on 150mm plates with 2.5μg/mL 6-TG. For plating efficiency, 500 PC3 cells were seeded on a 6-well plate. Cells were stained with crystal violet and counted. Relative mutation frequency was computed by the number of 6-TG resistant clones relative to the plating efficiency.

### Non-homologous end joining (NHEJ) DNA repair assays

These assays were done with the NHEJ reporter cells derived from KCL-22 cells carrying stably integrated EJ5-GFP total NHEJ construct and tetracycline-inducible I-SceI expression system as previously described [5]. For I-SceI induction, cells were treated with 1μg/mL doxycycline (Thermo-Fisher) for 72 h. Cells were co-stained with 4′,6-diamidino-2-phenylindole (DAPI) for live/dead selection and live cells were gated for GFP analysis. Flow cytometry was performed on a LSR Fortessa flow cytometer (BD Biosciences) and data analysis was carried out using FlowJo.

### Statistical analysis

Student's T-Tests were utilized to calculate the differences between experimental variable and control. Unless stated otherwise, bar graphs represent means ± standard error the mean (SEM). Statistical significance is called when p-value is less than 0.05.

## SUPPLEMENTARY MATERIALS FIGURES


