# Supplementary material for: SIRT1 and LSD1 competitively regulate KU70 functions in DNA repair and mutation acquisition in cancer cells
Source: Oncotarget. 2016 Jun 30;7(31):50195–214. doi: 10.18632/oncotarget.10328 (PMC5226577; doi:10.18632/oncotarget.10328)
Supplement: Supplementary file 1 [file oncotarget-07-50195-s001.pdf]

SIRT1 and LSD1 competitively regulate KU70 functions in DNA repair and mutation acquisition in cancer cells

SUPPLEMENTARY FIGURES

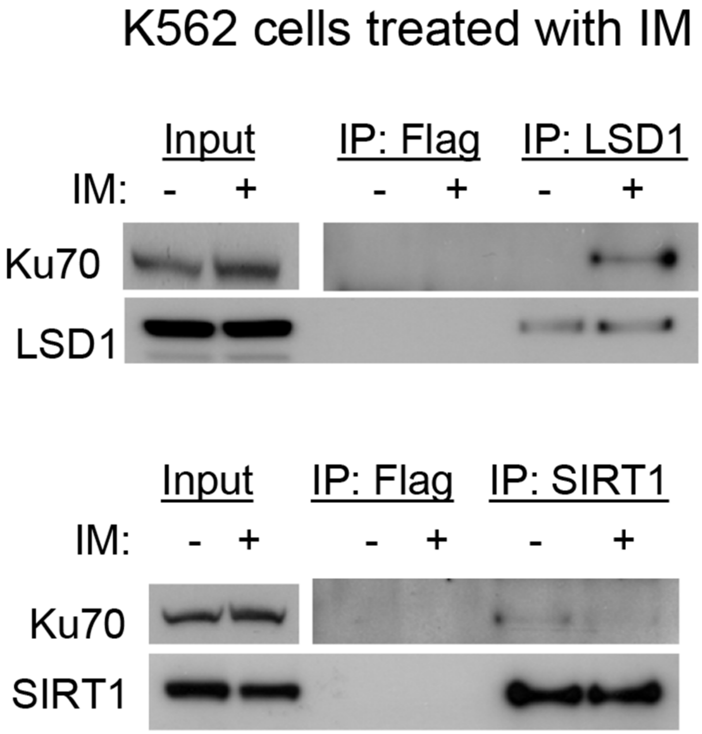

Supplementary Figure S1: Switching of LSD1 and SIRT1 binding with KU70 in response to stress. LSD1 and SIRT1 binding with KU70 in K562 CML cells in response to IM. Immunoblots of co-IP using antibodies for LSD1 (upper) and SIRT1 (lower) in K562 cells untreated or treated with 0.5μM Imatinib for 24 hours.

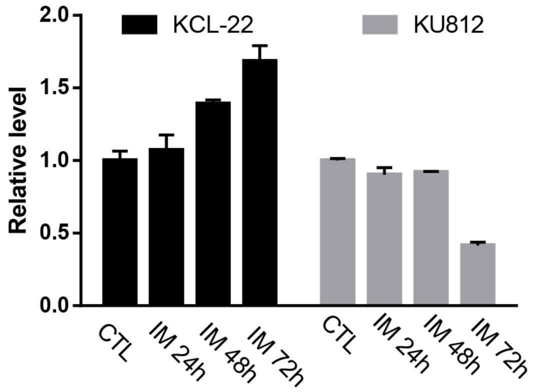

BCR-ABL mRNA expression normalized to control (CTL)

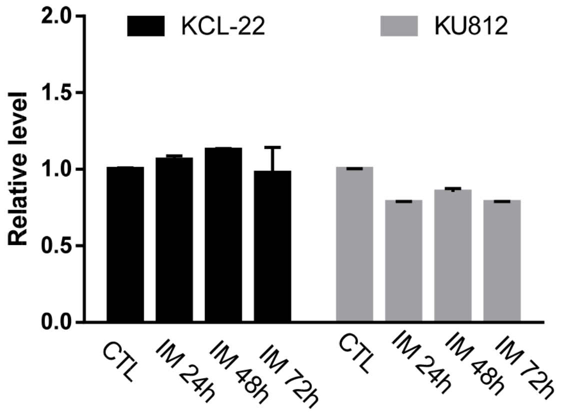

β-actin mRNA expression normalized to control (CTL)

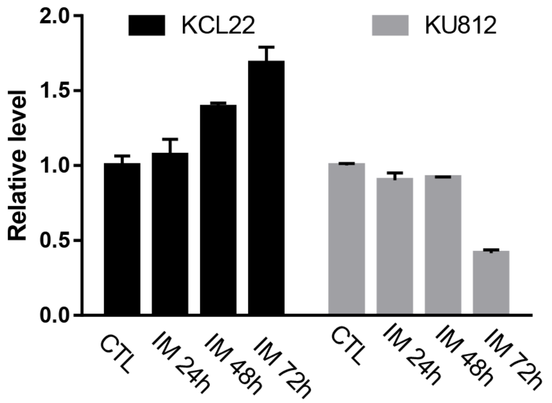

BCR-ABL mRNA expression normalized to control and to β-actin

Supplementary Figure S2: Analysis of BCR-ABL mRNA expression in KCL-22 and KU812 cells after 2.5 and 0.5 µM IM treatment, respectively, for the indicated time points.

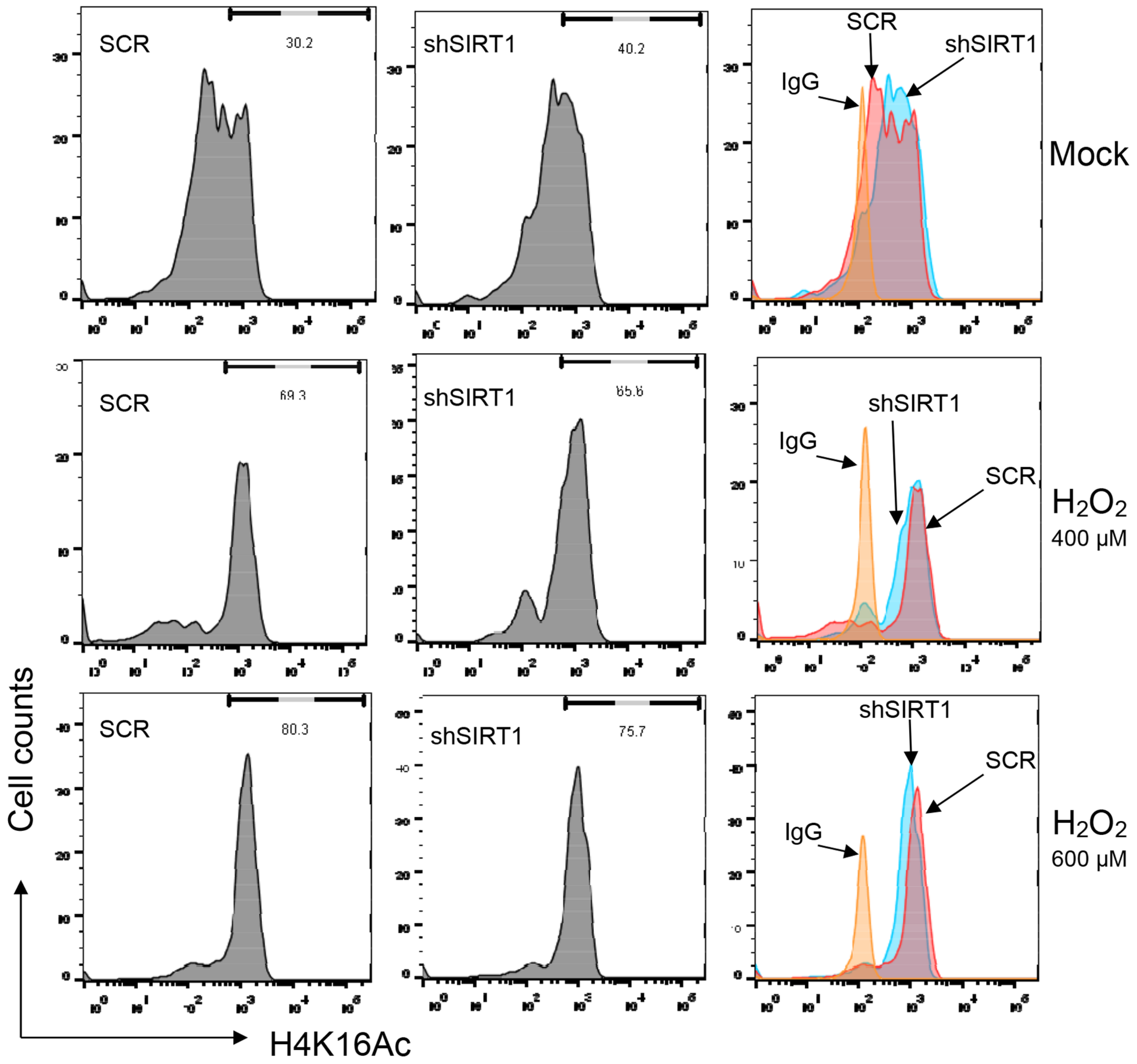

Supplementary Figure S3: Flow cytometry analysis of H4K16Ac in K562 cells after treatment with H_2O_2 for 2 h. Overlay charts were shown in color.

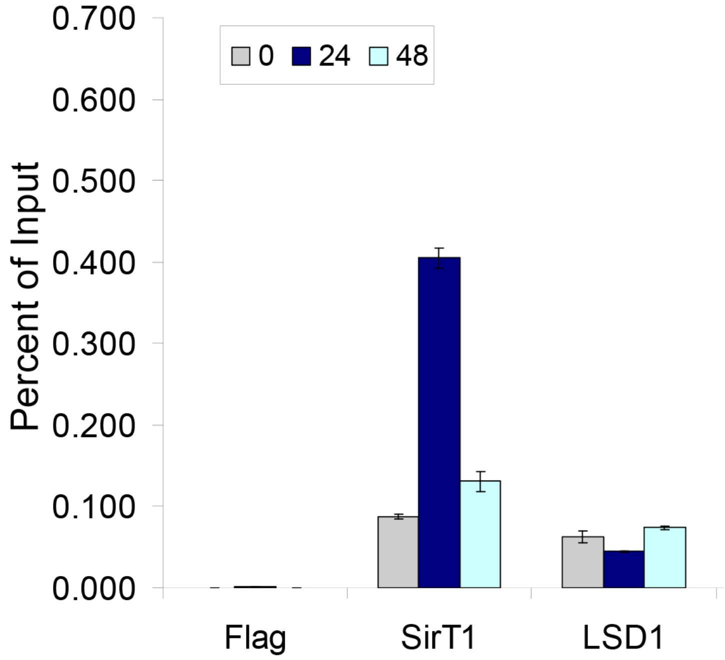

Supplementary Figure S4: Real-time ChIP-PCR analysis of recruitment of LSD1 and SIRT1 onto ABL exon 4 after 2.5 µM IM treatment for 24 and 48 h. Flag antibody was used as a negative control.

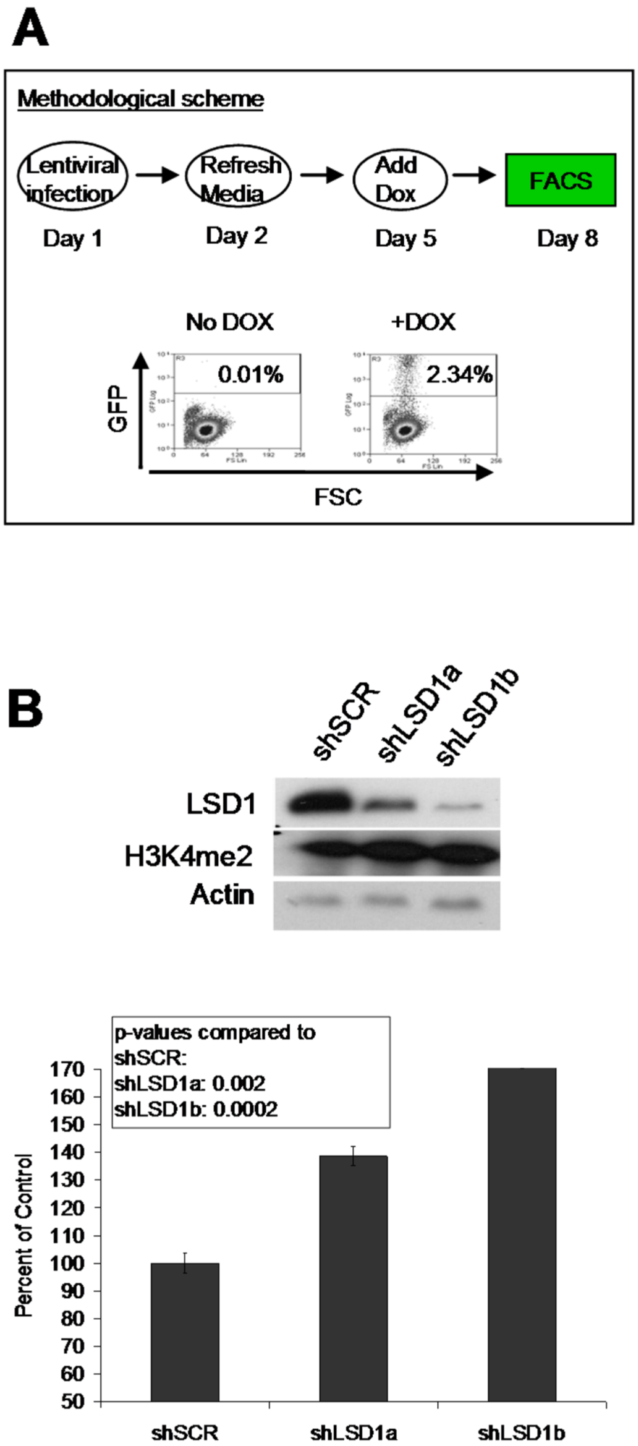

Supplementary Figure S5: NHEJ reporter assay with LSD1 knockdown. A. A schema showing KCL-22 cell based NHEJ reporter assay with Dox-inducible I-SceI expression. LSD1 was knocked down using lentiviral shRNA in the reporter cells, followed by treatment with doxycycline (DOX) for three days. GFP expressing cells as a result of NHEJ was then quantified by flow cytometry. **B.** NHEJ repair assay in the reporter cells with two sets of LSD1 knockdown shRNAs, shLSD1 a and b compared to scrambled shRNA (shSCR). Top panel shows immunoblots of these NHEJ repair cells using antibodies as indicated. Bottom panel shows bar graphs of means and SEM of GFP positive cells. Scrambled shRNA cells were considered at 100% and experimental group cells were normalized to shSCR. Student's T-tests were performed to compare each LSD1 shRNA sample to shSCR.

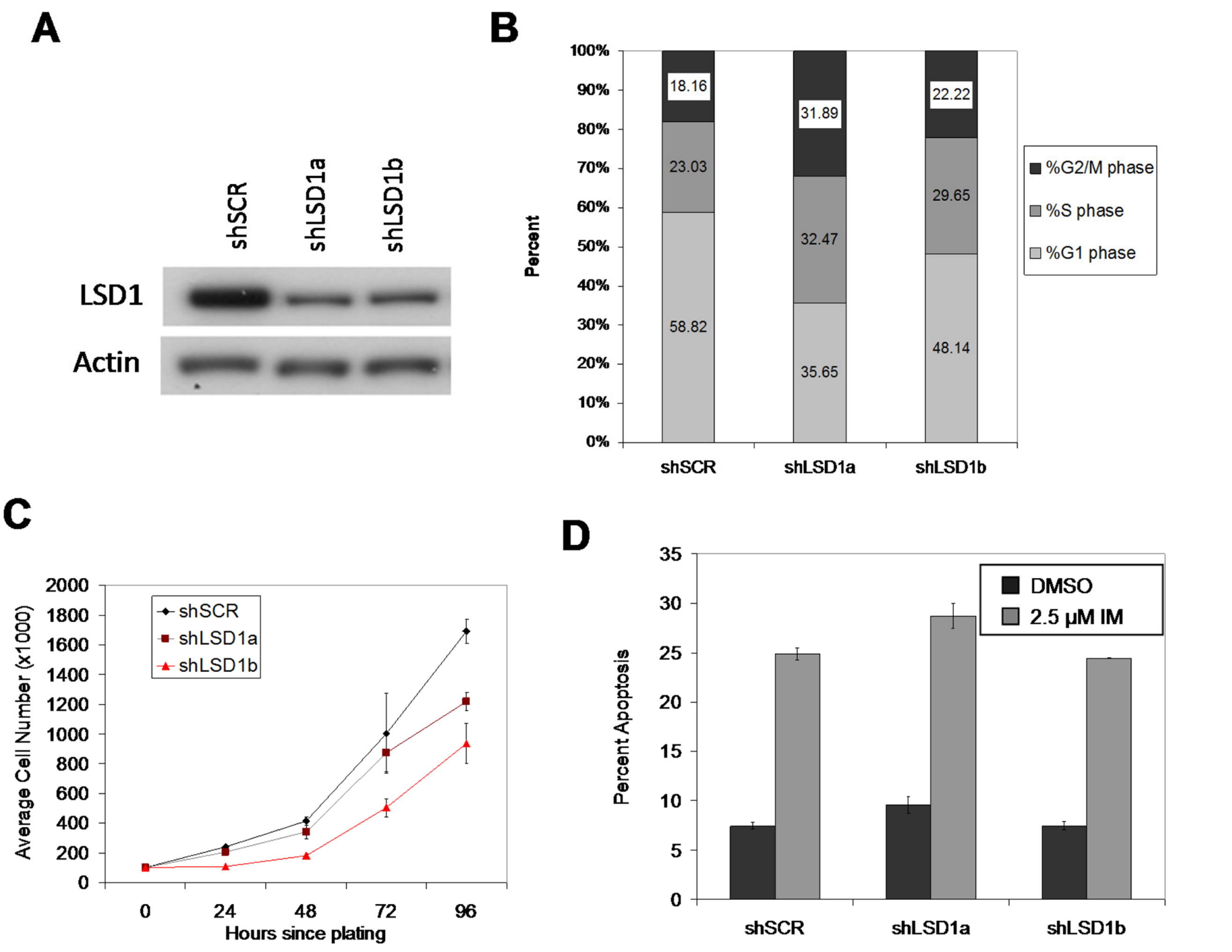

Supplementary Figure S6: LSD1 knockdown in KCL-22 cells altered cell cycle, proliferation and apoptosis. A. Western blot of LSD1 knockdown in KCL-22 cells. **B.** Cell cycle analysis of LSD1 or Scrambled control knockdown in KCL-22 cells. LSD1 knockdown cell exhibited the increased G2/M arrest by Modfit's cell cycle quantification. **C.** Growth curves of KCL-22 cells with LSD1 knockdown. **D.** Apoptosis of KCL-22 cells with LSD1 or shSCR knockdown in the presence and absence of imatinib. Bar graphs represented mean percent apoptosis as defined by Annexin V positive cells after 72 hours of mock or imatinib treatment.

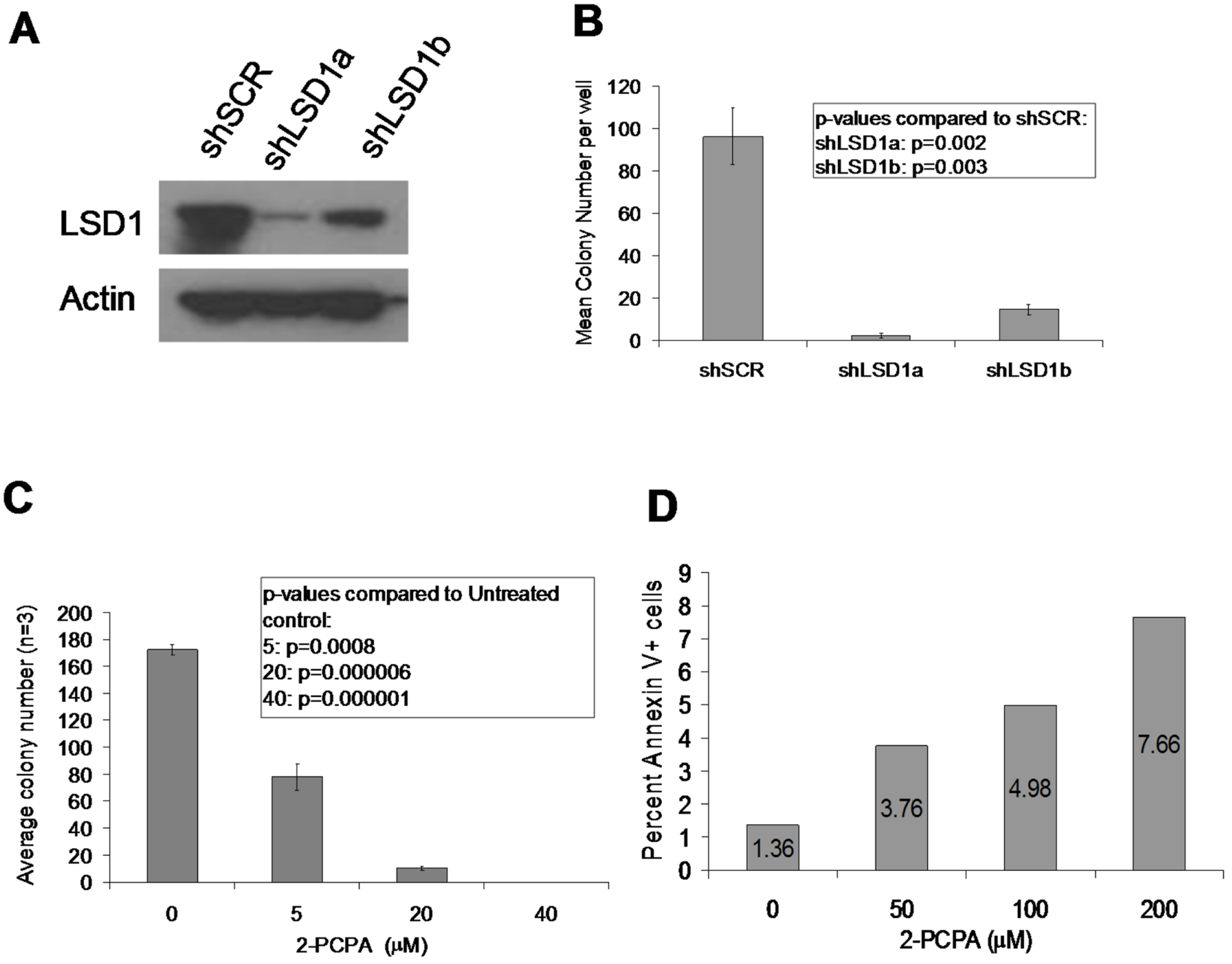

Supplementary Figure S7: Effects of LSD1 inhibition in TF-1 cells. A. Western blot showing LSD1 knockdown in TF-1 cells. **B.** Knockdown of LSD1 in TF-1 cells significantly reduced the anchorage-independent soft agar growth. **C.** Treatment with 2-PCPA inhibited soft agar growth of TF-1 cells. The cells were pre-treated with 2-PCPA for 72 hours before they were plated on soft agar with the drug at the concentrations indicated. **D.** Apoptosis induction of TF-1 cells treated with 2-PCPA for 72 hours as determined by annexin V positive cells by flow Cytometry.

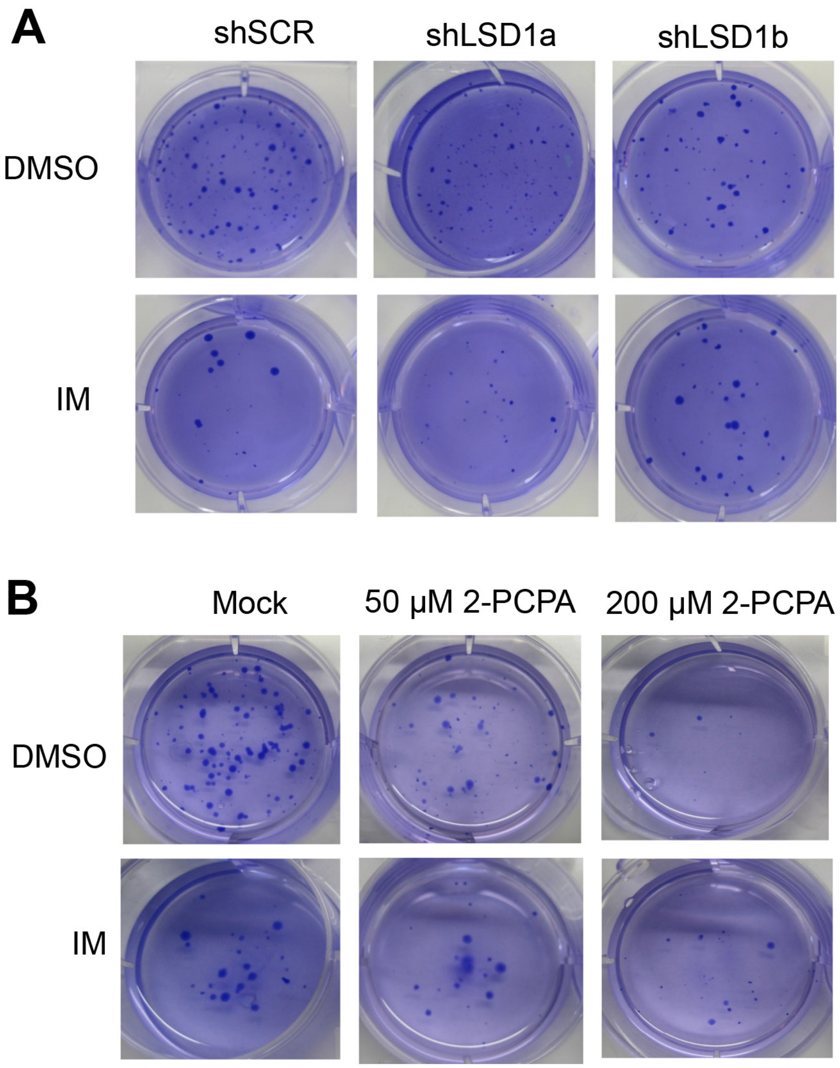

Supplementary Figure S8: Effects of LSD1 inhibition on IM-resistant BCR-ABL mutations. Representative images for IM-resistant colony formation of KCL-22 cells following shLSD1 knockdown **A.** or 2-PCPA **B.**

A

	Exon 6	Exon 4
shSCR		
untreated	0/2	ND
IM(2.5μM)	6/6 T315I	ND
shLSD1 #a		
untreated	0/2	ND
IM (2.5μM)	6/6 T315I	ND
shLSD1 #b		
untreated	0/2	0/2
IM (2.5μM)	1/6 T315I	5/6 Y253H
IM + DMSO	4/4 T315I	ND
IM + 50 μM 2-PCPA	4/4 T315I	ND
IM + 200 μM 2-PCPA	3/4 T315I	ND

ND: not done. shSCR: scrambled shRNA

B

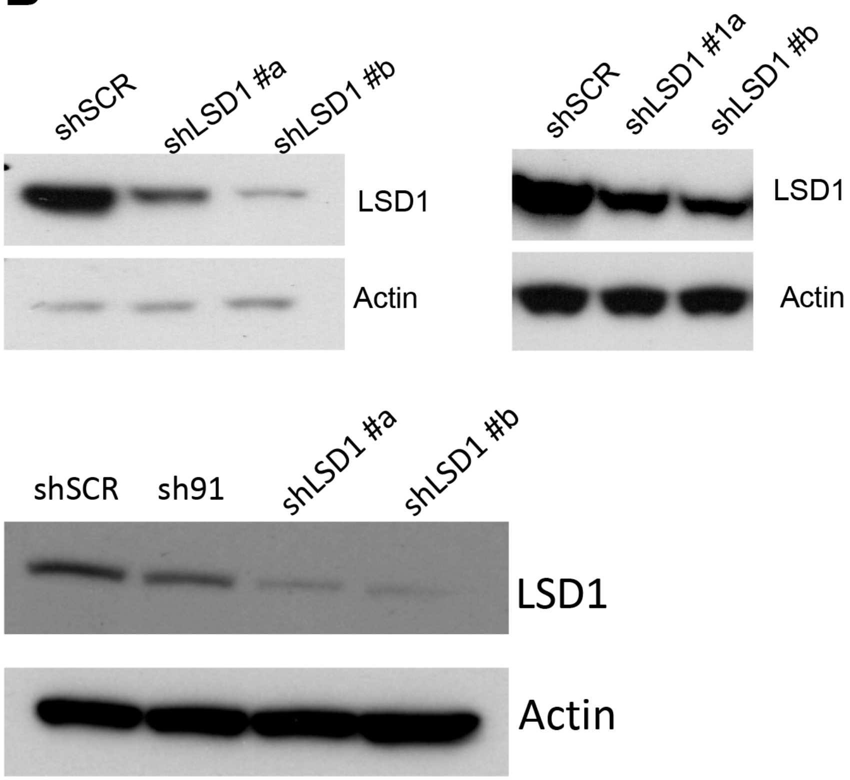

Supplementary Figure S9: BCR-ABL mutation analysis after LSD1 inhibition. A. Most IM-resistant clones analyzed had acquired T315I mutation, except clones with LSD1 knockdown by shLSD1 #b. **B.** Representative Western blots from three independent experiments showing that shLSD1 #b tended to have better knockdown efficiency than shLSD1 #a.

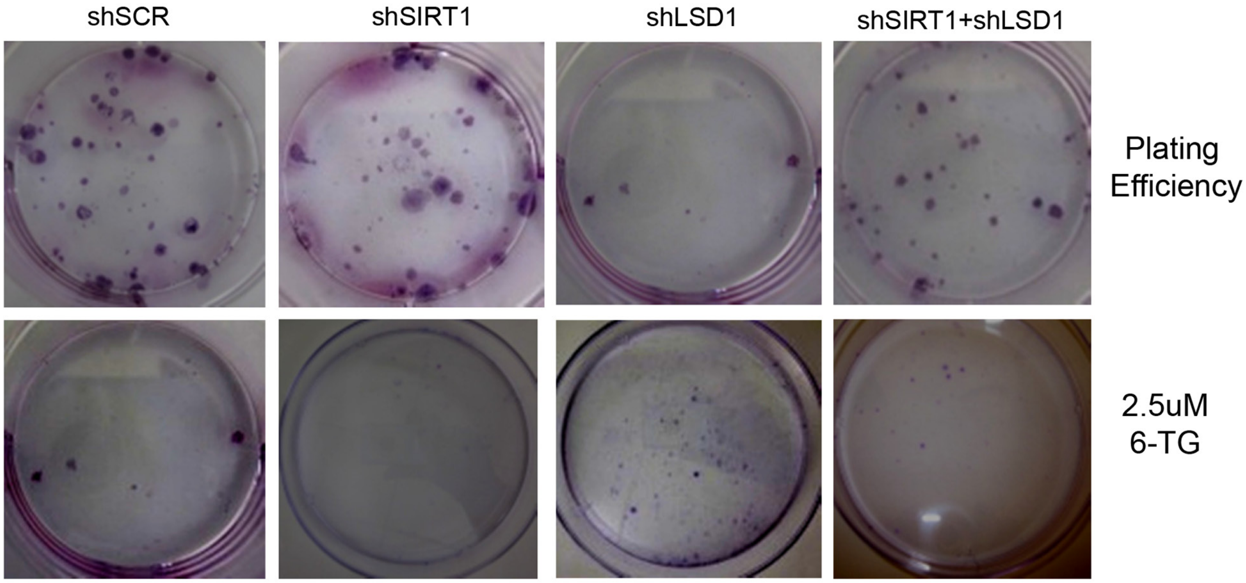

Supplementary Figure S10: Effects of LSD1 and SIRT1 knockdown on HPRT in mutations. Representative images of plaque formation of prostate cancer PC3 cells upon shSCR, LSD1, SIRT1 or combined LSD1/SIRT1 knockdown in the absence (plating efficiency, 500 cells/well) or presence of 6-TG, as described in Figure 9.

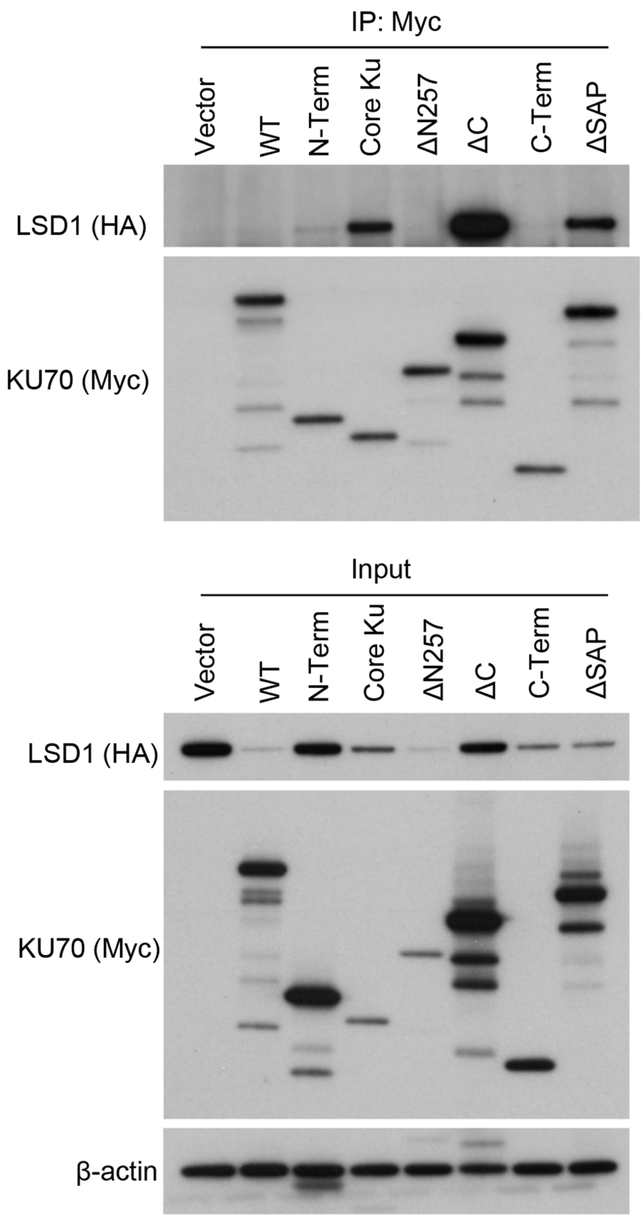

Supplementary Figure S11: Mapping of KU70 domains for interaction with LSD1. An independent analysis was carried out for mapping LSD1/KU70 interaction as in Figure 10B. Increased variation of HA-LSD1 levels and increased KU70 degradation were noticed when they were co-transfected. Precise reasons for these changes were unknown.
